# Bottom-up assembly of viral replication cycles

**DOI:** 10.1038/s41467-022-33661-7

**Published:** 2022-11-02

**Authors:** Oskar Staufer, Gösta Gantner, Ilia Platzman, Klaus Tanner, Imre Berger, Joachim P. Spatz

**Affiliations:** 1 Max Planck-Bristol Center for Minimal Biology, University of Bristol, 1 Tankard’s Close, Bristol, BS8 1TD UK; 2 Kennedy Institute of Rheumatology, University of Oxford, Roosevelt Drive, OX3 7FY UK; 3 Max Planck School Matter to Life, Jahnstraße 29, 69120 Heidelberg, Germany; 4 Theological Seminary, Heidelberg University, Kisselgasse 1, 69117 Heidelberg, Germany; 5 Department for Cellular Biophysics, Max Planck Institute for Medical Research, Jahnstraße 29, 69120 Heidelberg, Germany; 6 Institute for Molecular Systems Engineering and Advanced Materials (IMSEAM), University of Heidelberg, Im Neuenheimer Feld 225, 69120 Heidelberg, Germany; 7 School of Biochemistry, Biomedical Sciences, University of Bristol, 1 Tankard’s Close, Bristol, BS8 1TD UK; 8 Bristol Synthetic Biology Centre BrisSynBio, University of Bristol, 4 Tyndall Ave, Bristol, BS8 1TQ UK

**Keywords:** Biophysical methods, SARS-CoV-2

## Abstract

Bottom-up synthetic biology provides new means to understand living matter by constructing minimal life-like systems. This principle can also be applied to study infectious diseases. Here we summarize approaches and ethical considerations for the bottom-up assembly of viral replication cycles.

## Abstract (web summary)

Synthetic biology encompasses engineering approaches to either redesign biological organisms with new molecular abilities or build up organism from the bottom to improve our understanding of life. Here, the authors summarize current approaches that can be applied for the synthetic engineering of viral replication cycles and discuss the potential DURC risk associated with such bottom-up approaches in infectious disease.

## Bottom-up engineering of life-like systems

Bottom-up synthetic biology has opened up new avenues for the construction of artificial systems that recreate the structure and function of living cells. New fundamental knowledge about the mechanisms underlaying life, such as the physical principles of cell division^1^, cellular motility^2^, cell communication^3^ and morphogenesis^4^ has been achieved by the application of in vitro reconstitution approaches. In the present context, we define bottom-up synthetic biology as a field that strives to construct minimal life-like materials by bottom-up reconstitution of cellular phenomena in vitro^5^. Towards this, biological and artificial building blocks are applied to create molecular structures that feature functions inherent to life. Historically, and driven by its emergence from the physical sciences, the field has focused on the construction of synthetic cells that recapitulate the constitutional characteristics of natural cells (e.g. metabolism^6^, division^1^, evolution^7^, information processing^8^, compartmentalization^9^). In its essence, bottom-up synthetic biology redefines the algorithm of conventional biological research, that follows an observe-describe-understand concept, by studying living systems in a design-build-understand manner. This learning-by-building strategy is empowered by reducing the molecular complexity of cellular phenomena, allowing for a step-wise deconstruction of life´s most fundamental processes. Along this line of research, several new tools have been developed to engineer artificial cells, for instance microfluidic technologies to build and manipulate lipid vesicles serving as synthetic cell scaffolds, and advanced genetic engineering strategies to create synthetic cells that exchange information with their environment^8,10^. In this regard, the ever-increasing molecular toolbox for in vitro reconstitution of cellular phenomena has greatly accelerated progress in the field^11^, bringing the ultimate goal, crafting of a truly living synthetic system, within reach.

Apart from research efforts directed towards unrevealing the fundamental principles of cellular life forms, several studies have provided an initial demonstration of how the reductionistic design-build-understand approach can be applied to biomedical research objectives^12–15^. In recent years, studies based on minimal biological systems have brought forward synthetic cells designed for therapeutic purposes. These include synthetic cells capable of controlled production and release of insulin upon exposure to increased glucose concentrations in vivo^16^ and synthetic cells that autonomously produce therapeutic proteins within tumors^17^. These advances showcase the potential of bottom-up synthetic biology in biomedical applications and inform research that could empower completely new therapeutic agents. Moreover, this also demonstrates how artificial biological systems can be applied to decipher molecular mechanisms underlying disease states. Importantly, such engineering strategies are based on a fundamental principle of synthetic biology: modularity. Researchers in bottom-up synthetic biology, design individual cellular modules (e.g. compartments^18^, cytoskeletons^19^ or phase-separated organelles^20^) that can be combined in almost a plug-and-play manner. This enables breaking down biological questions into addressable subsets while allowing for the sequential deconstruction of individual molecular mechanisms. As such, studies based on a bottom-up strategy are especially powerful to deconvolute molecular mechanisms of disease processes that occur in a sequential and modular manner. The reconstitution of viral infection cycles is a prime example of a systematic dissection by bottom-up synthetic biology principles.

In fact, earlier studies have demonstrated how viruses, or parts thereof, can be assembled bottom-up and this ambitious approach has been recently transferred into the realm of SARS-CoV-2 research^21,22^. Intriguingly, in vitro reconstitution of the SARS-CoV-2 viral envelope and integration of the spike glycoprotein on lipid vehicles revealed how inflammatory fatty acids are exploited to trigger a molecular switch that couples local inflammation states to SARS-CoV-2 infectivity^23–25^. These efforts are not only driven by the implementation of synthetic biology in biomedicine but also mark the beginning of a first bottom-up assembled viral infection cycle. Bottom-up construction of fully artificial, custom-designed viral replication systems holds great promise to systematically dissect and decipher the sequential process during viral infection, replication, propagation, and transmission. This approach could be particularly valuable for SARS-CoV-2, the causative agent of the ongoing global pandemic, as resolving the sequential process associated with intracellular viral replication, could complement the ongoing elucidation of immunological aspects in COVID-19 and uncover novel mechanisms that can be potentially targeted to interfere with viral propagation. Based on recent progress in synthetic cell engineering, researchers in the field now dispose of a wide variety of molecular tools that could be combined and adapted for bottom-up engineering of a synthetic viral replication cycle recapitulating in a defined and sequential, and therefore controllable way, the corresponding process of the live virus.

## A modular approach towards engineering of synthetic viral infection cycles

**Fig. 1:**
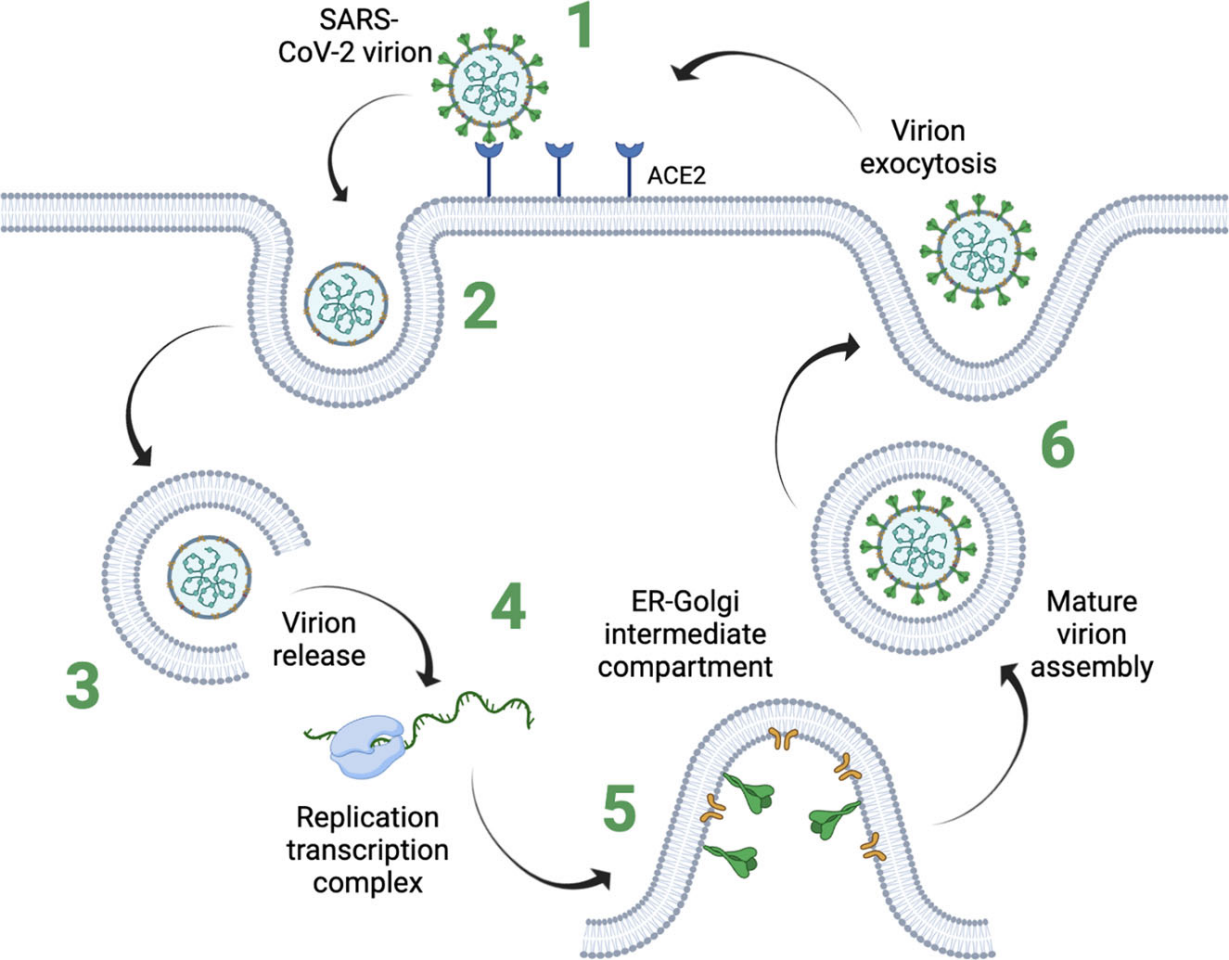
Viral replication cycle. The SARS-CoV-2 infection cycle is depicted in a schematic view, highlighting the sequential and modular nature of the replication process. For each step of the viral replication cycle, viruses have evolved specific molecular modules that are efficiently compressed into single virion particles.

Towards the bottom-up assembly of a compartmentalized virus-like structure capable of intracellular replication, certain essential features of the virions of choice need to be considered for the design process: Virions have evolved as highly efficient carriers of genetic information that have mastered the reprogramming of host cells for viral propagation. As inherent infectious agents, viruses exploit the molecular machinery of host cells to produce and propagate viral particles. The viral infection process itself is modular, and therefore ideally suited to be tackled by synthetic biology approaches^26–28^ (Fig. 1). Generally, the individual elements associated with intracellular viral replication cycles vary between virus species but typically include six defined sequential modules:

1. A module that allows identification of and binding to target cells by molecular recognition patterns (e.g. the receptor-binding domain (RBD) in the SARS-CoV-2 spike glycoprotein).
2. A structural module and co-stimulatory factors initiate the uptake of the virions by the host cell (e.g. the viral envelope of the SARS-CoV-2 virion).
3. A module that facilitates the release of viral genetic information into the cell cytoplasm or nucleus (e.g. the fusion domain of the SARS-CoV-2 spike glycoprotein).
4. A module that initiates the engagement of the host cell transcription-translation machinery in order to synthesize additional genomic copies and viral capsids.
5. A module that regulates the reprogramming of the host cell to facilitate viral capsid assembly (e.g. the formation of subcellular viral factories by the ER–Golgi intermediate compartment in SARS-CoV-2-infected cells).
6. A module that allows for the controlled exocytosis of correctly assembled virions.

Importantly, viruses have evolved towards a precise molecular orchestration of the individual modules and virions have been optimized by evolution to compress all required functionality into a single structure characterized by the lowest achievable complexity. This evolution-driven reduction of complexity is paralleled in bottom-up synthetic biology research, underscoring the value of this approach to studying viral replication. For instance, in the case of SARS-CoV-2, several functionalities (i.e. modules) have been merged into a single molecule, the spike glycoprotein that realizes receptor recognition, cellular uptake, and release of genetic information. Similarly, the SARS-CoV-2 genome encompasses multiple overlapping open reading frames, providing a highly efficient compression of genetic information. In this context, an important boundary condition for a bottom-up assembled viral replication cycle is the need for the genetic inscription of all structural and functional modules. This genetic information will need to be readable for the cellular machinery and allow for efficient production of new synthetic virion particles, mimicking exactly the process of a live virus. This also restricts the usage of non-natural building blocks, such as synthetic lipids, as these will need to be supplemented and processed by the host cells. At the same time, however, such exogenic functional elements might rationally be incorporated into the design in order to limit the propagation of synthetic viruses (such as nutrient limitation in bacterial strain culture) for biosafety purposes. Such biorthogonal switches would not only allow to cap off the synthetic replication cycle but also tune the temporal dynamics of the system when interfaced with living cells. In this regard, two fundamentally different scenarios and engineering strategies will need to be developed and combined. Firstly, a strategy for in vitro assembly of the initial synthetic viral particles. If necessary, this process can be performed under non-physiological conditions and supported by molecular engineering technologies such as microfluidics or organic synthesis of nanoparticles. The central purpose of these first-generation synthetic virions will be the initial transduction of host cells (including modules 1–3). Secondly, the synthetic virions generated will need to be capable of producing second-generation virions (modules 4–6), assembled in the intracellular environment, limiting the applicable synthesis tools, and building blocks to those provided by the cellular machinery.

A synthetic cycle should be designed under consideration of the natural mechanisms for viral replication to enable drawing biomedically relevant conclusions. However, the reductionistic approach pursued will require simplification and abstraction of certain modules, providing more controllable and quantitative means for virology as compared to studies based on natural viruses. Achieving a true integration of all required functional modules, within the boundaries of a particle of the lowest possible complexity, is potentially the most ambitious task for the bottom-up assembly of a synthetic viral replication cycle. For this, individual modules need to be designed with compatible chemistry and maintain functionality in the intracellular and extracellular environment. Although the unification of individual viral functionalities into a single particle structure is a challenge in itself, several technologies that allow engineering the synthetic replicates of the modules detailed above have been presented:

### Module 1

The main scaffolding element of membrane-enclosed virions are lipids that form from the host cell into a unilamellar lipid membrane around the densely packed virus genome. As compartmentalization is a central objective of bottom-up synthetic biology, a variety of technologies have been previously developed to precisely form and manipulate viral envelope-like lipid vesicles. For instance, liposome technologies have been adopted to construct artificial mimetics of several virus species^29^. Moreover, there are biorthogonal methods available for the controlled incorporation of recombinant viral proteins into these vesicles^30^. Liposomes have not only been functionalized with class I viral fusion proteins, but also with other targeting moieties that can direct liposome interactions with the cells of choice^31^. Therefore, an array of technologies is already available for the controlled assembly of synthetic virus scaffolding elements with cell-specific tropism.

### Module 2

For intracellular uptake, viruses exploit a combination of effects based on molecular self-assembly and receptor-mediated endocytosis^32^. Several studies have demonstrated that virus-like particles with a similar size to natural viruses can induce uptake by target cells via passive, entropy-driven membrane bending and receptor clustering^33^. Therefore, bottom-up assembled viruses will need to be optimized for receptor avidity by tuning the ligand density on their surface. In this way, efficient intracellular uptake could be rapidly achieved without the need for further complexity-increasing building blocks^32^.

### Module 3

As genetic information-carrying vehicles, virions are highly efficient in releasing their genetic cargo into cells. In fact, viruses have served as tools for drug delivery, aiming to achieve efficient integration of genomic elements into cells for therapeutic purposes. So far, even the most sophisticated artificial drug delivery platforms do not achieve the transduction efficiency and targeting-specificity of natural viruses. Therefore, most gene-therapy approaches are currently still based on viral vector systems. However, several molecular designs for the intracellular release of cargo from liposomal carriers have been adopted from viral blueprints. For instance, synthetic peptides mimicking the fusogenic properties of viral fusion proteins have been engineered on virus-like particles to achieve endosomal release of the cargo^34^. Other approaches exploit the intrinsic fusogenic abilities of viral envelope proteins, although recombinant versions of such proteins tend to display instability in solution. To overcome the stability limitations, hemagglutinin-like mechanisms have been mimicked by DNA nanotechnology, which allows proximity ligation of membranes^35^. However, fusogenic peptides provide a clear advantage over synthetic compounds as they can be genetically encoded for synthetic viral replication cycles. The peptides could potentially be tethered to the synthetic virus envelope via palmitoylation, inspired by the anchoring mechanism of the influenza fusion protein haemagglutinin^36^. Altogether, by taking inspiration from drug delivery technologies, fusogenic modules for synthetic virus replication are readily available and a controlled release of the genetic viral cargo can be achieved for synthetic viral replication cycles.

### Module 4

With the advancement of genetic engineering methods, viral genomes can be readily produced by recombinant DNA technology. Large viral genomes, such as SARS-CoV, horsepox, and baculoviruses, have been designed and sequentially assembled for gene therapy purposes^37–39^. Moreover, in the context of SARS-CoV-2, strategies to reverse engineer complete viral genomes from subgenomic fragments have been presented^40^. Based on this, tailored chemically synthetized viral genomes could be incorporated into virion-like particles, although the mere size of viral genomes will represent an additional challenge that needs to be tackled by advanced encapsulation technologies (such as those based on microfluidics^41^) for initial liposome assembly and programmed intracellular packing (see module 5). Likely, strategies to reduce the immunogenicity and increase the stability of artificial RNA genomes will be included, for example by incorporating pseudouridine nucleotides^42^. By incorporation of appropriate translational elements including ribosome binding sites and regulatory sequences, the intracellular transcription–translation machinery could be highjacked to produce second-generation genome copies and structural virion proteins. The proposed approach mainly aims to bottom-up assemble viral phenotypes (e.g. virions) that incorporate functional but artificial copies of viral genomes. The genomic element could either encompass the full genetic information found in the virus to be mimicked, or only retain those elements strictly necessary to achieve viral replication. Ideally, a viral particle could be designed that is able to replicate within cells without requiring genetic elements, although basing such a complex process only on self-assembly appears challenging. In order to minimize the genetic payload, single ORFs could be incorporated or a full genome with overlapping ORFs, synthesized by recombinant technologies, could be utilized.

### Module 5

From a molecular system engineering perspective, a module that achieves reprogramming of the cellular machinery and formation of intracellular viral factories that produce correctly assembled virions is presumably the most challenging. In this regard, exploiting the intrinsic mechanisms that would lead to the formation of viral factories, cellular replication networks, and lipid droplets is the most feasible approach as active engineering approaches could be circumvented^43–45^. Recent data suggest that intrinsically disordered domains in viral proteins (e.g. non-structural proteins 1 and 2) and host proteins initiate or sustain the formation of phase-separated organelles that serve as viral factories promoting the self-assembly of SARS-CoV-2 virion fragments^46^. Exploiting the natural function of non-structural SARS-CoV-2 proteins to engineer a synthetic replication cycle is a promising strategy as it may allow simultaneously blocking the translation of host mRNAs and enabling the formation of virion factories^47^. However, while designing a replication module on the basis of the cellular machinery allows one to closely model natural virus replication, this strategy compromises the quantitative and controllable characteristics of the overall system. For the initial benchmarking of this module, the bottom-up approach could be combined with a more top-down focused strategy based on in vitro transcription/translation systems from cell lysates. In this way, large-scale screenings in protocell systems could allow for targeted optimization of the conditions required for viral factory formation.

### Module 6

Cellular products are subjected to constant degradation and recycling at homeostatic conditions. However, newly assembled lipid-enveloped viral particles are able to escape from this autophagic process by promoting the directed sorting of virions into multivesicular bodies for side-directed exocytosis. Intracellular sorting of cargo for vesicle packing and endocytosis is based on a complex but coordinated interplay between supramolecular machines (e.g., the endosomal sorting complex required for transport, ESCRT) that localize specific cargos along the Golgi–ER-membrane axis. This machinery is highjacked by viruses for assembly and release of virions for future infection cycles^48^. In order to engineer this process in a controlled, tunable, and quantitative manner, modules inspired by extracellular vesicle (EV) engineering technologies could be applied. EVs have been linked to the evolution and maturation of viruses and have also been shown to play a critical role in SARS-CoV-2 infection^49,50^. Efforts directed towards the engineering of artificial EVs and specific loading of transgenic cargo into these cell-derived liposomes resulted in a rich toolbox of genetic engineering strategies that allow for programmable sorting of proteins and nucleic acids into multivesicular bodies for control packing into EVs. Specific genetically encoded EV sorting peptides derived from endogenous EV proteins (e.g., lysosome-associated membrane protein 2b, platelet-derived growth factor receptor, or tetraspanins) could be fused to proteins of interest in order to induce directed loading into virions during a synthetic viral replication cycle^51^. For sorting of nucleic acid components, methods based on the phase separation characteristics of EV sorting proteins (e.g., YBX1) could be repurposed to load RNA genomes into synthetic virions^52^. Furthermore, viral genomes could be tagged with specific EV-sorting sequences that by nature promote the inclusion of RNAs into EVs. Specific loading strategies of this kind could be quantitatively tuned by regulating the sumoylation of the sorting proteins^53^. The proposed engineering strategy would allow to the exploitation of the natural relationship between EVs and enveloped viruses, providing a potentially efficient instrument to regulate synthetic virion exocytosis.

## Ethical considerations of synthetic viral replication cycles

Technological progress is driven by ethical goals and technology aims to improve the quality of life for all people. Engineers developed codes of conduct that document this ethical orientation^54^. According to such written ethos, engineers should respect principles of sustainability, safety, health, and welfare of the public^55^. The research focused on bottom-up engineering of synthetic life-like systems, alike conventional engineering, is not value-free^56^. The suggested modularization approach, to create a synthetic virus replication cycle, is linked to the hope to mitigate some crucial risks associated with traditional virus research and is ultimately motivated by developing research tools for public benefits, such as new vaccines and gene therapy technologies.

In the 2010s, research on viruses attracted public attention due to genetic engineering efforts on influenza A virus subtypes (H1N1 / H5N1)^57,58^. Inspired by a top-down engineering strategy, research activities of concern included gain-of-function experiments that manipulated infectious agents, enhancing or generating potential for pandemic development. There was public concern that these engineered viruses would spread accidentally or be misused for terrorist or military purposes. Subsequently, several statements by scientific committees and research policy decisions emerged^59,60^. A moratorium in the US stopped public funding for such gain-of-function research on the influenza virus, severe acute respiratory syndrome (SARS) virus, and the Middle East respiratory syndrome (MERS) virus from 2014 to 2017^61–63^. This triggered a controversial discussion on ethical guidelines for the publication of security-relevant information^60,64,65^. The recent SARS-CoV-2 pandemic has brought these concerns back to the attention of the scientific community and alerted the wider public^66,67^.

In contrast to previous top-down genetic engineering approaches, the bottom-up engineering of synthetic viral replication cycles promises to minimize key risks, as a bottom-up design implicates a reduction of complexity, and synthetic viral replication cycles ideally only encompass properties that are absolutely necessary. This risk minimization by design would facilitate a high level of control over the engineered system. Along this line, several approaches have been developed to study the molecular mechanisms underlying viral infection, host immune response, and evolution of viruses. Some of the most extensively used tools include pseudoviruses and genetic engineering of viral genomes. Pseudoviruses are viral particles that do not display a relevant health threat to humans (e.g. based on lentiviruses) but incorporate molecular features of the virus under study. Pseudoviruses are especially valuable for analysis of host humoral immunity, e.g. in antibody-based neutralization studies. Moreover, they have been applied for several decades and a large body of experimental protocols and biosafety/biosecurity assessments exist. However, these systems do not allow to recapitulate of the biophysical properties of a target virus in full, a significant disadvantage that limits the translation of the obtained findings. Further, genetic engineering of viruses, although in parts challenging, has proven to be a powerful approach to studying viral infections, as it allows the assessment of viral genome organization and gene regulation. Moreover, with the help of viral genome engineering, gain-of-function (GoF) experiments can be performed by introducing recombinant DNA fragments and genes into viral genomes. However, as GoF experiments on human pathogenic viruses display a significant health threat, frequently revised regulatory mechanisms are in place to assess research work with unpredictable but most likely dangerous outcomes. In this context, the bottom-up assembly of viral replication cycles can be classified as a complementary technology. While the concept can be viewed as a GoF approach to introduce functionality into assembles of molecules instead of viral genomes, it mitigates the risk of uncontrolled viral replication by providing a high degree of molecular control. The complementary nature of the proposed approach allows to create molecular systems that recreate specific functional and structural features of viruses that cannot be obtained with conventional tools. For instance, it allows us to define and tune the density of fusion proteins present on the enveloping membrane, highlighting that in the bottom-up approach safety is mostly provided by degrees of control. However, as a novel approach, and in contrast to more established tools, bottom-up assembly of viral replication cycles lacks a significant amount of empiric knowledge that could be applied for risk assessment.

Research on bottom-up assembled viruses must deal with risks, either resulting from the research activity itself (*biosafety issues*) or regarding the use and misuse of the produced knowledge (*biosecurity issues*).

### Biosafety

In most expectations, artificial viral replication cycles appear to be harmless as long as they do not interact with natural systems. In the case of incubation with living cells, risk mitigation tools, such as the use of obligatory exogenous agents for synthetic virion replication, could be established to prevent the uncontrolled iterative replication of the designed systems outside of laboratory conditions. With such “safety bars”, foreseeable risks could be minimized to a greater extent. However, findings and biological insights reached with synthetic replication cycles are scope-limited, unless they are integrated into experimental setups with higher biological relevance (e.g. cells and animals). Conditions that are “more natural” and equipped with fewer safety mechanisms could increase the significance and impact of the research but need to be strictly balanced with the associated risk factors. In this context, research activities on potentially harmful and infectious agents must be conducted under appropriate biosafety precautions (e.g. laboratories with different biosafety levels)^68,69^. Different molecular safety bars could be designed to warrant the safe employment of each of the proposed modules. Specifically, the use of artificial promoters^70^ to drive gene expression in module 4, could be applied to specifically induce expression of the artificial genomes specifically within target cells. Such a genetically inscribed safety bar would display high resilience as an inherent design feature of the system. However, over several experimental cycles, genetic recombination could compromise the stability of such a genetic tool. Another safety bar to regulate and limit uncontrolled iterations between modules 6 and 1 could include encoding fusion incompetent release proteins. In this way, mature virions could be produced by the full completion of one viral cycle but unintended replication could be limited. It could be expected that such a safety bar shows high persistence in the designed system, as the spontaneous evolution of a fusion-competent form would require a series of mutations that would not be selected for under culture conditions. Further safety bars might include the deletion of glycosylation sites in viral proteins and other mechanisms of viral immune evasion. In this way, rapid clearance by the immune system would increase the biosafety level. Eventually, in order to maximize biosafety, a combination of several safety bars should be applied to compensate for the possible failure of single mechanisms in individual modules. This could be complemented by routine quality checks in experimental setups that include long-time frame experimental analysis, e.g. by sequencing the genomes present in a culture repeatedly. Therefore, we propose the following risk assessment scheme to categorize biosafety aspects in synthetic virus research:

- Low risk (low complexity): “safety bars” + research on isolated synthetic virus or individual modules, to be performed under biosafety level 1 condition.
- Medium risk (medium complexity): “safety bars” + synthetic virus is inserted into natural systems or a combination of two or more consecutive modules, to be performed under biosafety level 2 conditions.
- Higher risk (high complexity): “safety bars” + synthetic virus is inserted into natural systems with a set of modules that would allow full replication, to be performed under biosafety level 3 conditions.

Matching risk levels with laboratory biosafety levels not only offers elevated safety standards for research by increasing the protective measures (e.g. personal protective equipment and advanced safety instrumentation) but also provides extended documentation and surveillance protocols. Moreover, it offers a practical solution since BSL standards are well established across research institutions and associated regulations are in place. Nonetheless, as research risks might increase by interfacing synthetic viral replication cycles with other engineering strategies of limited predictive value (e.g. top-down genetic engineering of host cells), the proposed scheme only serves as initial benchmarking. The molecular design strategy can reduce the risk, but this does not imply that this approach is risk-free and that the security bar mechanism in place should not lead to misjudgment of the potential hazards. Therefore, such emerging bottom-up technologies should also be managed according to established safety regulations. In some instances, however, the current regulatory mechanisms might not prove sufficient, e.g. in the case that specific hazards are still unknown today. In such cases, institutions and involved stakeholders are required to commit to and adapt existing measures in order to mitigate associated risks. Ideally, the actors involved should develop a moral awareness that claims to oversee the consequences of their actions to the best of their knowledge. This moral awareness should be encouraged by scientific institutions and advisory comities. It also refers to fundamental principles such as sustainability, justice, peace, and human rights^71^. Research funders are also in a position to impose risk-minimizing conditions. In addition, there are national and international regulatory measures to protect the public^72^.

### Biosecurity

Regarding biosecurity issues, Dual Use Research of Concern (DURC)^73^ needs to be carefully considered within the bottom-up framework. For instance, targeted optimization of viral replication cycles is possible and could be legitimized for synthetic virus approaches. The suggested modularization approach, to create synthetic virus replication cycles, aims to mitigate some crucial risks associated with conventional research on natural viruses and is ultimately motivated by developing research tools for public benefits. However, with good reason, “engineering of viruses and viral delivery systems” is listed by the Fast Track Action Subcommittee on Critical and Emerging Technologies of the White House. The committee lists those “advanced technologies that are potentially significant to U.S. national security”^74^. This classification refers to the precautionary principle, which includes, but is not limited to, the question if technology has intolerable biosecurity risks^75,76^. Synthetic virus research implies technological knowledge that involves serious potential risks, some of which are known but most of which are yet unknown. In particular, emerging technologies in the field of virus research are partially associated with dystopian and catastrophic concerns^77^. The following remarks argue that specified risk assessment is needed based on the significant uncertainties regarding the proposed research concepts on synthetic viral replication cycles. We ask for risk assessment, which includes iterative monitoring mechanisms and integrates public consultations and concerns. Such a monitoring mechanism should evaluate biosafety and biosecurity issues of synthetic viruses while in progress and inform media, political actors, and members of political representative bodies. The aim must be to identify and mitigate risks from the very beginning.

Similar to the National Science Advisory Board for Biosecurity (NSABB, USA) the German Commission for Biosecurity (ZKBS) assesses current developments in the field of synthetic biology and makes its findings available to ministries, the parliament, and the public. The commission summarizes that current research on synthetic biology does not pose any risks to biosafety/biosecurity, either in Germany or worldwide, other than those already assessed with the help of the German Genetic Engineering Act and other international regulations for “conventional” genetic modifications^78^. However, this appraisal could change if it were to become possible to create synthetic viruses, which feature functional replication systems. The possibility of misuse (pathogens with pandemic potential) of this technology has led to a variety of security measures that could contain new regulations by the government. In Germany, there was a controversial debate on legal regulation measures, which was also reflected in the German Bundestag. Eventually, it was decided to retain the governance within the scientific community, informed by expert committees in the involved research institutions^79^. In accordance with the Recommendations for Handling Security-Relevant Research by the German Research Foundation (DFG) and the German National Academy of Sciences Leopoldina, the risk assessment comprises the following modules: Risk analysis that aims to minimize identified risks, documentation and communication risks, evaluation of proposed publications, and training of the staff involved^80^. A central module was the establishment of local commissions for ethics in security-related research (KEFs) at German research institutions^81^. The central task of these commissions is to identify possible risks, such as dual-use problems, at a very early stage and to propose suitable measures to minimize them. In individual cases, this examination may also lead to the decision, that “a high-risk project only being carried out at a later point in time, following a research moratorium, or perhaps not at all, even when the project is not prohibited by law”^80^. The Joint Committee on the Handling of Security-Relevant Research by the DFG and the Leopoldina advises the local KEFs (see for example the “Key questions for the ethical assessment of security-relevant research”^82^), documents the safety-relevant risks identified, functions as a platform to share experience in preventing risks and oversees the publication of the results. Importantly, this also applies to synthetic virus research. The experts involved should steadily review synthetic virus technologies in order to immediately report security-related hazards. The focus on biosecurity issues should not distract from the fact that there is also a need for governance in the field of biosafety^83^.

The actors involved also include scientists as authors of peer-reviewed publications and scientific publishers who may disclose security-related knowledge. However, biosecurity issues have yet not been the focus of published protocols or research results. The publication of the de novo assembly of SARS-CoV-2 genomes triggered discussions about necessary guidelines regarding security-relevant publications^84^. Members of the Engineering Biology Research Consortium (EBRC) recommend that the review and publication process needs to implement control mechanism to deal more seriously with safety and security issues: “The outcomes of the author, reviewer, and editor surveys should be used as a basis for discussion on minimizing publication risks. In some cases, additional safety and/or security experts may need to be engaged, and it may be valuable for authors to discuss security concerns that may result from publication with research institutions, funders, and (rarely) appropriate governmental officials”^85^.

What counts for security-relevant aspects of emerging technologies in general, applies to GoF research of pathogens in particular: It must establish an iterative, easily revisable risk assessment and risk management strategy. From a molecular perspective, the approach proposed here is also a GoF approach, which needs ethical and legal expertise from the beginning. On an international level, several guidelines and rules have been proposed and implemented^86–89^. At national levels, mechanisms for regulation have been adopted, for example, the HHS P3CO framework in the US^90^ and existing NIH guidelines are applicable, at least in part, to the research outlined in this comment^91^. Of note, these regulations are not considered strict enough for a larger scientific community and the Research Service of the US Congress listed several legal possibilities to regulate even more restrictively^92^. The extent to which GoF research at the molecular level should also be subject to stricter requirements should be part of an ongoing debate.

Apart from an unintended spread of engineered pathogenic viruses, one of the most serious biosecurity risks is the misuse of this technology. Mindful of biowarfare and bioterrorism, it must be carefully considered whether safety-relevant research results can be published in full. It must be prevented that synthetic viruses that are created through published knowledge to misuse these pathogens as weapons. This also concerns the filing of patents. Against the background of potential benefits for medical care, such preventive measures should not disproportionately hinder scientific exchange and technological progress^93,94^. These mechanisms are part of an permanent monitoring process that characterizes a responsible scientific culture within a “web of prevention”^95^. Responsible scientists have to acknowledge legal requirements, even though responsible science goes beyond legal regulations alone. Every scientist is also morally obliged to identify and mitigate risks in a reflexive and adaptive system of soft law regulation and self-governance^96^. This process could also make new national and international legal requirements necessary if the creation of artificial viruses will be feasible. Furthermore, the role of intelligence services is part of the web of prevention, which needs to be highlighted to detect threats that affect public health, the economy, and (national) security^97^. In this case, careful trade-offs must be made between freedom of research and safety obligations to the public. Of note, virus research will need to be increasingly understood in the global context that takes place under different national legal regimes^98^. The exchange of security-relevant data and research results can collide with existing export regulations^99,100^. While a moratorium or even a ban on synthetic virus research as the ultima ratio is conceivable, it is rather unlikely that such measures could be effectively enforced due to the number of researchers involved in different countries. Nevertheless, efforts must be intensified to establish international ethical and legal standards to guide security-related research^101,102^.

Possible misuse of the developed technologies could also arise from a do-it-yourself (DIY) research mentality. Such efforts largely evade regulatory and legal surveillance common to established research institutions. If successfully adopted, the proposed modular approach could reduce the tacit knowledge required to conduct experimental work on bottom-up assembled viral replication cycles. This in turn could result in biosecurity-relevant threats that could be exploited for the development of bioweapons. Codes of conduct and voluntary commitments already exist in the DIY communities^103,104^, which are the results of intensive reflection and debate^105^. The same legal requirements apply to DIY laboratories as to academic or industrial laboratories^106^. Therefore, also in the context of DIY research on bottom-up assembled viral replication cycles, increased awareness of security-relevant aspects should be encouraged and, if necessary, new legal measures should be implemented^107^.

Scientific data sharing and open-science efforts are particularly strong in the bioengineering field, wherefore publication bans would represent a remarkable restriction. Patents could also hinder scientific progress by restricting access to key technical information and licensing-based pay-walls^108–110^. Bottom-up assembled infection cycles may lead to patentable innovations that can be used for biomedical applications such as vaccine development. Especially regarding crises of a global scale, like the COVID-19 pandemic, it may be appropriate to tailor IP policies in terms of public health requirements^111^. In this view, various tools have been developed, such as patent pools or equitable licensing strategies of universities that are applicable to the bottom-up assembly of viruses^112–114^. Therefore, the ethical dimension of synthetic virion research involves the assessment of benefits and risks concerning patenting and licensing strategies.

Altogether, bottom-up engineering of viral replication promises improved risk management as the involved levels of complexity and associated risks can be controlled precisely. However, in cooperation with legal and administrative authorities, politicians, media, and the public, it is necessary to assess whether the existing laws are sufficient or whether new possibilities for technological misuse require new legal rules. Research that deals responsibly with the challenges outlined can only be guaranteed if the interconnected fields of action are clearly recognized. To attribute responsibility to the actors involved, it must be clear who performs what kind of research activity under which safety conditions and legal frameworks^115^. Of crucial importance for the acceptance of synthetic viruses, research will be risk management within ethics committees and other involved bodies from the scientific community as well as transparent communication. However, it is not possible to predict to which extent political decision-makers, NGOs, and media coverage will come to the same conclusions in their risk assessment, highlighting the importance of public trust in this discourse.

## Conclusions

In summary, for all of the presented modules, several biomimetic engineering strategies have been presented here that can be adopted for the design and bottom-up construction of synthetic viral replication cycles. However, the challenge becomes formidable when aiming to unify all solutions into a single structure, as current approaches to link individual modules are not compatible. For instance, while large viral genomes can be chemically synthesized, host intrinsic mechanisms that direct RNAs into vesicles are most efficient for sorting small RNA sequences such as microRNAs and mRNAs. Therefore, finding practical solutions to engineer a continuous and self-sustaining replication cycle will require creative approaches that might take further inspiration from artificial molecular engineering tools such as DNA nanotechnology or biorthogonal chemistry. At the same time, this will also need to be balanced with the need to mirror natural viral replication as close as possible to foster new application-focused insights into viral replication and the treatment of associated diseases.

Once these challenges are successfully overcome, a powerful new tool for virology could be created. In fact, virus biology has been revolutionized by the application of semi-synthetic virions earlier. For instance, pseudoviruses have enabled unpreceded quantitative insights into the mechanism underlying viral cell entry, immune evasion, and evolution in many viral diseases including SARS-CoV-2^116,117^. A fully synthetic viral replication cycle could additionally allow studying fundamental questions, such as the importance of the self-assembly process during virion maturation. Moreover, the physiochemical interaction between virions and host cells could be studied with a high spatial and temporal resolution by the use of artificial particles that closely mimic the biophysical properties of virions. With direct relevance for studies on SARS-CoV-2, the implementation of synthetic viral replication cycles could empower the identification of new functional mechanisms that might be targeted by dedicated antiviral treatments. Such mechanisms could include processes associated with phase separation during virus factory assembly, quantitative knowledge of which, and the role of cellular regulation, remain largely elusive. Furthermore, synthetic replication cycles could mature into a programmable tool to study mechanisms of cellular defense against virus-induced metabolic reprogramming and activation of immune evasive mechanisms. Ultimately, bottom-up assembly of viral replication cycles can bring forward a new generation of techniques that allow deconstructing the temporal dynamics underlying viral infection in mechanistic, molecular detail.
